# Platelet-Mediated NET Release Amplifies Coagulopathy and Drives Lung Pathology During Severe Influenza Infection

**DOI:** 10.3389/fimmu.2021.772859

**Published:** 2021-11-11

**Authors:** Seok-Joo Kim, Agostina Carestia, Braedon McDonald, Amanda Z. Zucoloto, Heidi Grosjean, Rachelle P. Davis, Madison Turk, Victor Naumenko, Silvio Antoniak, Nigel Mackman, Mohamed Sarjoon Abdul-Cader, Mohamed Faizal Abdul-Careem, Morley D. Hollenberg, Craig N. Jenne

**Affiliations:** ^1^ Department of Microbiology, Immunology, and Infectious Diseases, University of Calgary, Calgary, AB, Canada; ^2^ Department of Critical Care Medicine, University of Calgary, Calgary, AB, Canada; ^3^ UNC Blood Research Center, Department of Pathology and Laboratory Medicine, University of North Carolina at Chapel Hill, Chapel Hill, NC, United States; ^4^ UNC Blood Research Center, Department of Medicine, University of North Carolina at Chapel Hill, Chapel Hill, NC, United States; ^5^ Faculty of Veterinary Medicine, University of Calgary, Calgary, AB, Canada; ^6^ Department of Physiology and Pharmacology, University of Calgary, Calgary, AB, Canada

**Keywords:** influenza A virus, platelets, PAR4, thrombin, neutrophils, NETs, intravital microscopy (IVM)

## Abstract

The influenza A virus (IAV) causes a respiratory tract infection with approximately 10% of the population infected by the virus each year. Severe IAV infection is characterized by excessive inflammation and tissue pathology in the lungs. Platelet and neutrophil recruitment to the lung are involved in the pathogenesis of IAV, but the specific mechanisms involved have not been clarified. Using confocal intravital microscopy in a mouse model of IAV infection, we observed profound neutrophil recruitment, platelet aggregation, neutrophil extracellular trap (NET) production and thrombin activation within the lung microvasculature *in vivo*. Importantly, deficiency or antagonism of the protease-activated receptor 4 (PAR4) reduced platelet aggregation, NET production, and neutrophil recruitment. Critically, inhibition of thrombin or PAR4 protected mice from virus-induced lung tissue damage and edema. Together, these data imply thrombin-stimulated platelets play a critical role in the activation/recruitment of neutrophils, NET release and directly contribute to IAV pathogenesis in the lung.

## Introduction

Influenza is a common respiratory tract infection and a leading cause of death from infectious disease in United States (1). Influenza A virus (IAV) caused 50-100 million deaths during pandemic of 1918-1919, and continues to infect 10-20% of the population each year (2). Although adaptive immunity is essential for IAV clearance, innate immune cells also play key roles in viral control and IAV pathogenesis (3, 4). Critically, an overly robust innate immune response can lead to enhanced tissue damage, predisposing the patient to secondary infections (5, 6). Thus, though important for pathogen clearance, innate immunity also has the capacity to cause collateral damage and subsequent organ dysfunction if not properly regulated.

Much of the host inflammatory response is mediated by neutrophils, cells that are rapidly recruited to the lung in response to infection (7–9), representing one of the first, non-resident, immune responders against pathogens (10). Neutrophils mediate host-defense against a wide variety of invading microorganisms, possessing a broad arsenal of defense strategies, including release of neutrophil extracellular traps (NETs); highly charged mixtures of decondensed DNA, nuclear, and granular proteins. These structures entrap and kill pathogens and can neutralize viral particles (11).

During IAV infection, neutrophils have been shown to limit viral replication and disease progression (12, 13). However, this local increase in neutrophil recruitment may not be entirely beneficial as it can also inflict damage, exacerbating pulmonary inflammation through excessive NET release (14, 15). Platelets also play a significant role in host defense to infection by expressing immune receptors and immune effectors upon activation (16). During inflammation, platelets recognize, sequester and kill pathogens directly (17, 18), and regulate immune cell recruitment and activation (19). Platelet-neutrophil interaction is often required for cellular recruitment to infection sites, and studies have demonstrated that this interaction is crucial for NET release (20).

Further linking infection and platelets, IAV infection leads to increased tissue factor expression, thrombin generation and activation of the coagulation cascade (21). Importantly, dysregulation of coagulation has been associated with increased morbidity and mortality in other viral lung infections. Early data from the COVID-19 pandemic has indicated evidence of coagulopathy in the most severely affected patients and this systemic activation of coagulation is associated with end-organ damage and subsequent death (22–24). These observations highlight the need to understand the interplay between infection, inflammation, and coagulation.

Here, using high-resolution confocal intravital microscopy (IVM), we demonstrate that collaboration between thrombin, platelets, and NETs leads to enhanced inflammation and tissue damage during IAV infection. Using a fluorescent enzyme substrate and a PAR4 inhibitor, we demonstrate that thrombin, *via* platelet activation, is a principal driver of IAV-mediated lung pathogenesis.

## Materials and Methods

### Mice and Viruses

C57Bl/6 mice were purchased from Jackson Laboratories (Bar Harbor, ME, USA). CD41-/- (CD41YFP/YFP, yellow fluorescent protein [YFP] sequence knocked into the CD41 locus) mice were on a C57BL/6 background and were provided by Dr. K. McNagny (University of British Columbia). PAR4-/- mice on the C57Bl/6 background were generated as previously described (25). All mice were 7-10 weeks old and maintained in a specific pathogen-free environment at the University of Calgary. Influenza A virus strain A/PR/8/34 (PR8, H1N1) was grown in 10-day embryonated hen’s eggs by standard procedures and titrated on Madin-Darby canine kidney (MDCK) cells as described (26). All experimental animal protocols were approved by the University of Calgary Animal Care Committee in compliance with guidelines from the Canadian Council for Animal Care (Protocol AC18-0050).

### Infection and Treatment of Mice

Under isoflurane anesthesia, mice were challenged with intranasal (i.n.) PR8 infection (250 PFU unless otherwise indicated). Mice were weighed daily and assessed for visual signs of clinical disease. Animals that lost 25% of their original weight were removed from the study and euthanized. At day 1, day 3 or day 5 post-infection, IVM was conducted.

*In vivo* blockade of CD18 was achieved by intravenous (i.v.) administration of 100 µg of anti-CD18 (clone GAME-46, eBioscience, San Diego, CA, USA) 1 h prior to IAV infection. For *in vivo* PAR4 inhibition, 20 mg/kg of an antagonist peptide, TcY-NH2 (Tocris Bioscience, Bristol, UK) dissolved in 25 mM sterile HEPES, was administered intraperitoneally (i.p.) 20 min prior to IAV infection. Thrombin inhibitor, argatroban (Sigma; 10 mg/kg), was dissolved in DMSO and 100 μl weas delivered i.p. once 30 min before IAV infection or daily starting at day 3 of infection. DNase I (Roche; 2000 international units) was resuspended in water as per manufacturer instructions and delivered i.v. immediately before IAV infection and once a day for 4 days.

### Preparation of the Mouse Lung for Intravital Microscope

For microscopic visualization, we used a modification of the methodology proposed by Looney et al. (27). In brief, mice were anesthetized with ketamine (200 µg/kg; Bayer Animal Health, Toronto, Canada) and xylaxine (10 mg/kg; Bimeda-MTC, Cambridge, Canada) i.p. and the jugular vein was cannulated to permit the injection of fluorescently labeled antibodies and for the maintenance of anesthetic. A tracheotomy was performed and PE90 tubing was inserted into the incision site and secured with a silk suture. Following i.t. catheterization, mice were mechanically ventilated at 150 breaths/min with 120 μl of tidal volume using an Inspira ASV (Harvard Apparatus, Holliston, MA, USA). Mice were placed in the right lateral decubitus position, and the lung left lobe was exposed through removal of the overlying skin, fat, and sections of two to three anterior ribs. A thoracic suction window attached to a micromanipulator on the microscope stage was then placed into position and 20–25 mm Hg of suction was applied (Amvex Corporation, Richmond Hill, Canada) to gently immobilize the lung. The water-immersion objective (HC FLUOTAR L 25x/0.95 W VISIR) of resonant-scanning confocal microscope (TCS-SP8, Leica Microsystems, Concord, Canada) was lowered into place over the thoracic suction window. This microscope platform is equipped with multiple excitation lasers (405 nm, 488 nm, 552 nm, 647 nm), a tunable digital emission filter and 5 independent detectors [2 photomultiplier tubes (PMT), 3 hybrid HyD SP GaAsP detectors (HyD)] allowing for simultaneous imaging of up to 5 independent channels (Davis et al., 2020). Typical microscope settings were: for the 405 nm excitation laser (33.8% power), emission filter scanned from 410 to 468 nm (detector PMT1, gain 814.4V); for the 488 nm excitation laser (28% power), emission filter scanned from 501 to 544 nm (detector HyD2,gain 18.8%) and from 571 to 586 (detector HyD4, gain 35%); and for the 638 nm excitation laser (24.9% power), emission filter scanned from 656 to 696 nm (detector HyD5, gain 16.5%).

### Antibodies and Fluorescent Probes

For IVM, Brilliant Violet 421-conjugated anti-Ly6G (neutrophils; clone 1A8, BD Biosciences, San Diego, CA, USA, 1.6 μg), AlexaFluor 647- or PE-conjugated anti-CD49b (platelets; clone HMα2, BioLegend, San Diego, CA, USA, 1.6 μg), PE-conjugated anti-CD31 (endothelium; clone 390, Biolegend, 0.8 μg), goat anti-mouse histone H2Ax (clone M20, Santa Cruz Biotechnology, USA, 2 μg) was conjugated to AlexaFluor 555 and goat–anti-mouse neutrophil elastase (M18, 2 μg) was conjugated to AlexaFluor 647 using a labelling kit as per the manufacturer’s instructions (Life technologies, Carlsbad, CA, USA). Quantities and clones of antibodies used to label neutrophils and platelets have been previously optimized and demonstrated to have minimal impact on cellular recruitment and or clearance through mechanisms such as antibody-dependent cellular cytotoxicity (28, 29). Thrombin was visualized with 5-FAM/QXL^®^ 520 FRET substrate (SensoLyte^®^ 520 Thrombin Activity Assay Kit, AnaSpec, Inc., Fremont, CA, USA). FITC- or AF647-albumin was used to delineate the vasculature.

### Analysis of Resonant-Scanning Confocal Microscope-Acquired Images

In most experiments, anti-CD31 or albumin as a contrast agent was used to landmark lung structures and set imaging depth. Total numbers of neutrophils were counted for of 3-6 FOV per animal and averaged. Quantification of NETs, platelet aggregation and thrombin activity involved measurement of the total area of staining for each marker per FOV in a minimum of 3 FOV per animal. Detail analysis parameters were as described previously (30, 31). The number of platelet aggregates ≥10, 25, 50, or 100 µm^2^ were counted using the Analyze Particles function within ImageJ, as previously described (28).

### Immunohistochemistry

Infected mice were euthanized with CO_2_, lungs removed and immediately frozen in optimal cutting temperature (OCT) media. OCT-embedded frozen tissues were sectioned (7 μm), fixed in PFA 4%, blocked with PBS containing 1% donkey serum and 3% BSA, permeabilized with PBS/Triton 0.01% and stained with fluorescently-conjugated antibodies.

### Bronchoalveolar Lavage Fluid (BALF) and Lung Edema

Following euthanasia, a catheter was inserted into the trachea. BALF was collected by five washes of 1 mL of PBS containing 5mM EDTA and total cell numbers was determined using a hemocytometer. Lung edema was measured in separate animals. Lungs were removed, weighed, dried for 72 h at 65°C, and then reweighed in order to obtain the wet-to-dry tissue weight ratio as previously described (32).

### Myeloperoxidase (MPO) Activity

Lungs were homogenized in detergent (0.5% hexa-decyltrimethylammonium bromide buffer), and supernatant was reacted with hydrogen peroxide and a hydrogen donor, O-dianisidine dihydrochloride. Reaction was measured over time *via* a colored compound development using a spectrophotometer to determine MPO content (450 nm, 25°C; SpectraMax Plus 384, Molecular Devices, Sunnyvale, CA, USA).

### Flow Cytometry Analysis

Lung tissues were harvested and minced with scissors in an ice-cold HEPES solution and then were digested with collagenase D (2 mg/mL) and DNase (80 U/ml) for 30 min in 37°C. After the digestion, tissues were passed through a 70-μm filter and washed with HEPES supplemented with 2% FBS. Following centrifugation, erythrocytes were lysed with hypotonic buffer and the remaining leukocytes were washed in FACS buffer (PBS+5% fetal bovine serum+0.5 mM EDTA). Cells were blocked with an FcR-blocking antibody (clone 2.4g2) and stained for neutrophils (Ly6G), platelets (CD41), and markers of neutrophil activation (CD11b, clone M1/70 and PSGL-1, clone 2PH1) for 30 min on ice. The cells were washed and analyzed using an Attune NxT flowcytometer (Thermo Fisher Scientific).

### Quantification of Extracellular DNA

Complexes of DNA bound to histones were measured in plasma and BALF using commercially available cell death detection ELISAPLUS kit according to the manufacturer’s instructions. The calibration curve was constructed using a standard of nucleosomal DNA of a known concentration.

### Platelet Counts

Platelets were counted manually using a hemocytometer.

### Viral Load Assessment

MDCK cells were cultured in DMEM (10% FBS,1% Penicillin-Streptomycin) until confluent, seeded in 12-well plates and incubated at 37°C and 5% CO2 for 24 hours to generate a monolayer. Lungs were harvested 5 days post-infection, homogenized and used to produce ten-fold serial dilutions in PBS. MDCK monolayers were infected with 50μL of each dilution (1h at 37°C, 5% CO2) followed by addition of agar overlay. Plates were incubated (37°C, 5% CO2 48 h), washed and plaques visualized by staining with crystal violet for 15 minutes. Plaque-forming units (PFUs)/mL were calculated and converted into a log10 scale.

### Statistical Analysis

All results are presented as means ± SEM. Overall significance was examined by one-way analysis of variance with post-hoc Bonferroni test (for parametric data) or a Kruskal-Wallis with post-hoc Dunn’s test (for nonparametric data). Differences between the groups were considered statistically significant at a P < 0.05.

## Results

### IAV Induces Platelet Aggregation, Neutrophil Recruitment, and Inflammation in the Lung

**Figure 1:**
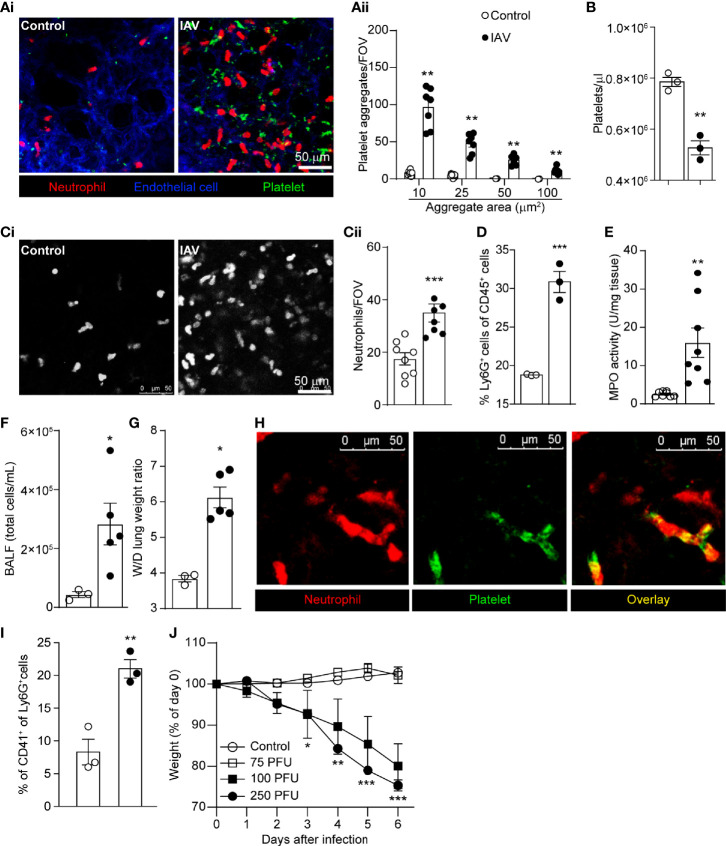
Intranasal IAV Infection of Mice Results in Platelet Aggregation, Neutrophil Recruitment, and Lung Edema. **(Ai)** Intravital visualization of platelet and neutrophils following administration of PBS or infection with IAV in the lung vasculature (neutrophils, red; platelets, green; endothelium, blue). **(Aii)** Quantification of platelet aggregates of the indicated sizes in PBS-treated mice or IAV-challenged mice. Values are shown as mean ± SEM, n=8 per group. **(B)** Peripheral blood platelet counts in control and IAV-infected mice. **(Ci)** Imaging (neutrophils, white) and **(Cii)** quantification of neutrophil recruitment within the lung in PBS-treated mice or IAV-challenged mice. Values are shown as mean ± SEM, n=6-8 per group. **(D)** Flow cytometric quantification of the percentage of neutrophils (Ly6G^+^ cells) in the lung. MPO activity **(E)**, BALF cell counts **(F)** and lung wet-to-dry tissue weight ratios **(G)** following IAV infection. Values are shown as mean ± SEM, n=3-8 per group. **(H)** Co-localization of neutrophil and platelet aggregates within the lung vasculature of a IAV infected animal (neutrophils, red; platelets, green). **(I)** Flow cytometric quantification of the percentage of neutrophils (Ly6G^+^ cells) that are interacting with platelets (CD41^+^) in the lung **(J)** Animal weight measurements following infection with indicated inoculums of IAV. *p < 0.05, **p < 0.01, ***p < 0.001 compared to the control group.

Previous reports indicated activation and aggregation of platelets within the lung vasculature worsen the severity of lung injury during viral infection (33–35). Moreover, platelets potentiate leukocyte recruitment to sites of infection (36) and neutrophils interact with damaged endothelium in areas rich in platelet aggregates (37). Direct interaction between platelets and neutrophils results in further neutrophil recruitment and activation (38). To better understand these dynamic cell-cell interactions within the intact lung, we utilized IVM (**Supplemental Figure 1**) to study a mouse model of severe IAV infection. Mice were challenged with 250 PFU IAV A/PR/8/34 (H1N1) i.n. and studied 5 days post-infection. This model of IAV infection results in all animals reaching the humane weight-loss threshold of 25% by day 6-7 post-infection. Although some impact on cell mobility may be expected following surgery to visualize and stabilize the lung, using IVM, we observed a marked increase in platelet accumulation and the formation of large platelet aggregates within the lung vasculature following IAV infection as compared to control animals (**Figure 1Ai**). Quantification of aggregates demonstrated a significant increase in both small (10 and 25 µm^2^) and large platelet aggregates (50 and 100 µm^2^) following IAV infection (**Figure 1Aii**). Moreover, IAV infected mice have significantly fewer circulating platelets (**Figure 1B**). Additionally, IAV induced profound neutrophil recruitment to the lung (**Figure 1C** and **Supplemental Video 1**). These IVM findings were confirmed by flow cytometry and by the detection of elevated MPO levels in the lungs of IAV infected animals (**Figures 1D, E**). Analysis of BALF showed IAV increased the number of cells within the bronchoalveolar space (**Figure 1F**). Finally, IAV induced a significant increase in wet-to-dry weight ratios of the lung (**Figure 1G**), a validated measure of overall tissue fluids (32). These results show IAV infection induces a rapid platelet and neutrophil recruitment to the lungs and subsequent pulmonary edema.

Platelets recruited to the infected lung were observed interacting with adherent neutrophils (**Figure 1H**) forming large, dynamic aggregates (**Supplemental Video 2**). By introducing a fluorescent contrast agent (labeled albumin) into circulation, we demonstrated that these aggregates are in the intravascular space (**Supplemental Figure 2**). These observed neutrophil-platelet interactions were confirmed by flow cytometry (**Figure 1I**). Additionally, severe IAV infection results in significant animal weight loss over the course of infection (**Figure 1J**), demonstrating this model of IAV infection induces significant disease and inflammation in the lung that is reflective of reports following severe IAV infection of patients (13, 14). Considering that the number of pulmonary vessels can differ depending on the depth of the observation region, neutrophil counts and platelet aggregates were analyzed by vessel area, and similar results were obtained (**Supplemental Figure 3**).

### Neutrophil Accumulation and Formation of Neutrophil-Platelet Aggregates Are Mediated by Integrins

Blockade of adhesion molecules, such as CD18 (β2 integrin), dramatically reduces platelet recruitment to the inflamed liver (28). Moreover, CD18 has been implicated in the binding of platelets to neutrophils (20), and this binding subsequently triggers neutrophil activation and migration (39). The integrin CD41/CD61 (GPIIb/IIIa), which is found solely on platelets, is responsible for the formation of fibrinogen bridges among platelets, facilitating platelet cohesion, aggregation, and subsequent thrombus growth (40, 41).

**Figure 2:**
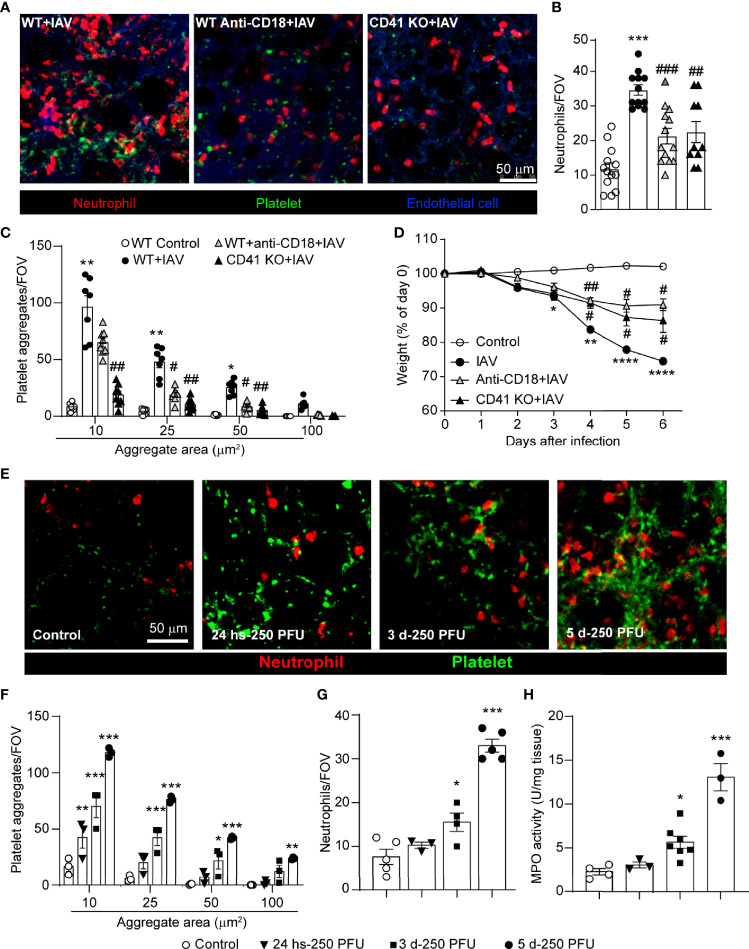
Dynamics of Virus-Induced Neutrophil Recruitment and Platelet Accumulation in the Lungs. **(A)** Intravital visualization of neutrophils and platelet aggregation in the lung vasculature 5d-post IAV-infection in animals with anti-CD18 blocking antibody (WT Anti-CD18+IAV) or CD41 deficiency (CD41 KO+IAV) (neutrophils, red; platelets, green; endothelium, blue). Quantification of neutrophils **(B)** and platelets **(C)** within the lung as measured by IVM. Values are the mean number of neutrophils per field of view (FOV) ± SEM, n=3-5 per group. **(D)** Animal weight measurements following infection with or without integrin blockade/deficiency. Values are shown as the mean of weights normalized to day 0 ± SEM, n=3-5 per group. **(E)** IVM of neutrophil recruitment and platelet aggregation in control, 24h-post IAV infection and 3d and 5d-post IAV infection (neutrophils, red; platelets, green). **(F)** Quantification of platelet aggregates of indicated sizes **(F)** and neutrophils **(G)** at indicated times post IAV infection. **(H)** MPO activity at indicated times post IAV infection. Values are the mean number of aggregates per field of view (FOV) ± SEM, n=3-5 per group. *p < 0.05, **p < 0.01, ***p < 0.001 ****p < 0.0001 compared to the control group; ^#^p < 0.05, ^##^p < 0.01, ^###^p < 0.001 compared to the IAV-infected group.

Based on these studies, we interrogated the role of these integrins in neutrophil and platelet accumulation in the lungs following viral challenge. A significant reduction in neutrophil recruitment and platelet aggregation in the lung microvasculature was observed following IAV infection in mice pretreated with an anti-CD18 blocking antibody (**Figures 2A**–**C**, **Supplemental Video 3**), a finding that was confirmed by flow cytometric analysis of the lung (**Supplemental Figure 4A** **).** Analysis of cellular behavior demonstrated that IAV infected mice have increased neutrophil diameter and decreased roundness, key markers of cellular activation (**Supplemental Figures 4B, C**). These results were confirmed by an elevated expression of CD11b, but interestingly not PSGL-1, by neutrophils isolated from the blood, lung and BALF (**Supplemental Figure 4D**). Although blockade of CD18 reduced overall neutrophil recruitment, no impact on cellular diameter (lengthening of the neutrophil) or cellular roundness (more pseudopod formation; **Supplemental Figures 4B, C**) was observed. Moreover, the increased neutrophil crawling observed following IAV infection was diminished by CD18 blockade (**Supplemental Figure 4E**) indicating CD18 was required for neutrophil motility but not for neutrophil activation. Targeting platelet-expressed integrins, CD41-deficient mice had less platelet accumulation in the lung after IAV infection than did wild-type mice (**Figure 2C**). Interestingly, we also observed reduced neutrophil recruitment to the lung in CD41-deficient mice following IAV infection (**Figure 2B**).

Although neutrophil recruitment often precedes platelet aggregation (28, 30), other studies have demonstrated a role for platelets in facilitating neutrophil recruitment to various vascular beds (42, 43). The phenotype observed in the CD41-deficient animals suggested a significant proportion of neutrophil recruitment to the lung following viral challenge is mediated by platelets. Moreover, CD41-deficiency also reduced both neutrophil activation and crawling (**Supplemental Figures 4B, C, E**) suggesting platelet aggregation was not only critical for neutrophil recruitment but also played a role in cellular activation and motility. Concomitant with this reduced cellular recruitment, an attenuation of weight loss after IAV infection was observed in the presence of CD18-blockade or CD41-deficiency (**Figure 2D**). This potential linkage between platelet integrins and neutrophil recruitment led us to investigate if platelet recruitment to the lung precedes neutrophil accumulation in severe IAV infection. Assessment of early cell recruitment (24h post-infection) reveals a clear and significant accumulation of platelets (**Figures 2E, F**) prior to observed increases in neutrophil numbers (**Figures 2E, G**). Furthermore, IVM and MPO assays revealed that neutrophil recruitment starts after 3 days of infection (**Figures 2G, H**). Importantly, this early role for platelets was only evident during severe IAV infection; infection with a lower inoculum of virus (25 PFU) failed to induce platelet accumulation in advance of neutrophil recruitment (**Supplemental Figure 5**).

### IAV Infection Induces the Release of NETs Within the Lung Vasculature

**Figure 3:**
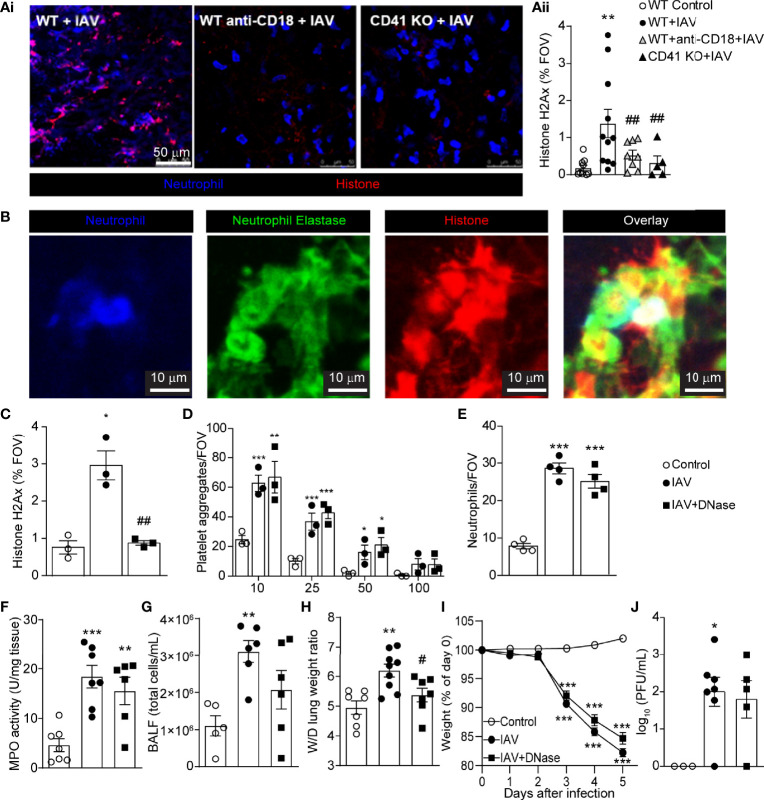
IAV Infection Induces the Release of NETs within the Lung Vasculature. **(Ai)** IVM imaging of NETs in IAV-infected animals pretreated with anti-CD18 blocking antibody or animals deficient for CD41 (neutrophils, blue; extracellular histone, red). **(Aii)** Quantification of the area of staining of extracellular histone. Values represent staining area as a % of the FOV and are presented as the mean+/- SEM. n=5-9 per group **(B)** Representative IVM imaging of the lung vasculature 5d post-IAV infection (neutrophils, blue; neutrophil elastase, green; extracellular histone, red). Quantification of histone H2Ax **(C),** platelet aggregates of indicated sizes **(D)** and neutrophils **(E)** measured by IVM in mice treated with DNase. Quantification of lung MPO activity **(F)**, total BALF cellularity **(G)**, and lung wet-to-dry tissue weight ratios **(H)** following IAV infection of control and DNase-treated animals. Animal body mass **(I)** and lung viral loads **(J)** following IAV infection of control and DNase-treated animals. Values represent the mean +/- SEM. n = 5-9 per group. *p < 0.05, **p < 0.01, ***p < 0.001 compared to the control group. ^#^p < 0.05, ^##^p < 0.01 compared to the IAV-infected group.

Neutrophil-platelet interactions drive NET production in response to several viral pathogens (14, 30, 44, 45). IVM analysis identified a significant increase in extracellular histone H2Ax in the lung following IAV infection (**Figure 3A**). Analysis of plasma and BALF by ELISA confirmed the presence of elevated extracellular histone levels following IAV infection (**Supplementary Figures 6A, B**). Blockade of CD18, or deficiency in CD41, significantly inhibited extracellular histone deposition in the lung (**Figure 3A**), demonstrating a role for platelet-neutrophil interaction in the release of extracellular DNA structures in response to IAV infection. As extracellular histone may correspond to both NETs and other extracellular structures (e.g. DNA released from cell lysis), we sought to confirm if histone staining corresponded to NETs in the lung vasculature following IAV infection. Immunostaining of IAV-infected lungs reveals extensive colocalization of staining of extracellular histone and NE (**Figure 3B**), markers that when found together are hallmarks of NETs. Moreover, quantification of extracellular NE staining by IVM confirmed a significant increase in this marker within the lungs following IAV infection (**Supplementary Figure 6C**).

NETs, if produced in an uncontrolled fashion can cause damage to the vasculature and associated tissues. To determine if NET production contributed to IAV-mediated lung pathology, mice were treated with DNase, an enzyme that cleaves and clears NETs and extracellular DNA structures (**Figure 3C**) but leaves recruited neutrophils and platelets unaltered. Breakdown of NETs and/or extracellular DNA did not affect total platelet aggregates (**Figure 3D**) or neutrophil recruitment to the lung, as measured by IVM (**Figure 3E**) and MPO activity (**Figure 3F**). Removal of NETs and/or extracellular DNA did reduce BALF cellularity (**Figure 3G**) and led to a significant decrease in the wet-to-dry weight ratio of lung tissue (**Figure 3H**), suggesting NETs and/or extracellular DNA contribute to localized lung damage. Despite this reduction in localize lung pathology, DNase treatment did not prevent animal weight loss or impact viral clearance (**Figures 3I, J**), a finding that is in agreement with an earlier study in PAD4-deficient mice (46). These results indicate that clearance of NETs and/or extracellular DNA does not change the overall outcome for the animal.

### Thrombin Mediates Platelet Aggregation and NET Release

**Figure 4:**
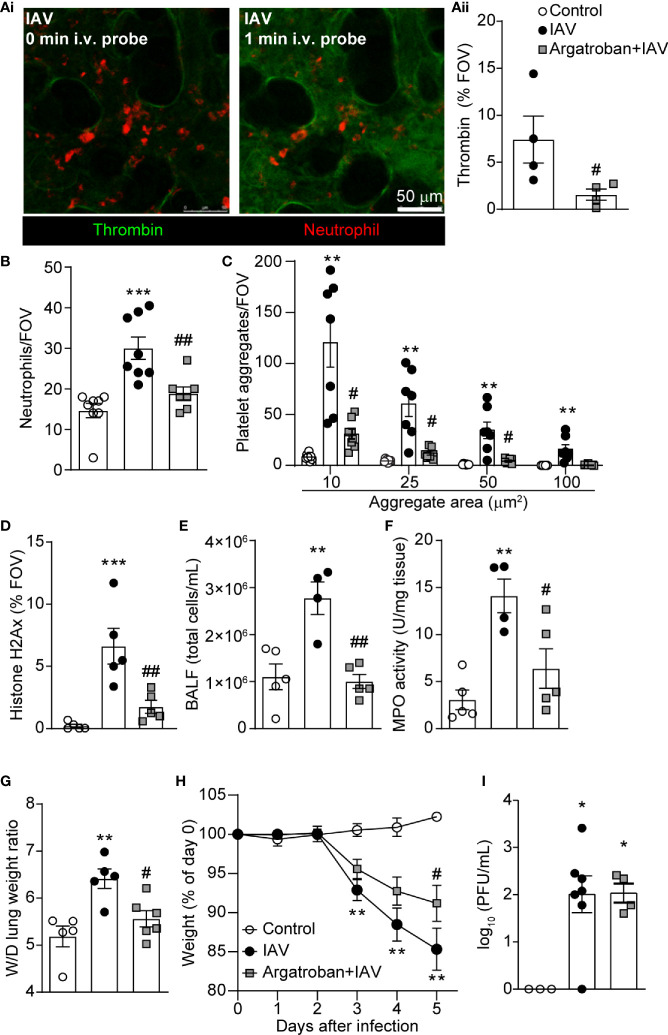
Thrombin Mediates Platelet Aggregation and NET Release within the Lung Vasculature during IAV Infection. **(Ai)** Representative IVM images of thrombin probe cleavage in the mouse lung 5d-post IAV-infection at the time of probe infusion (0 min, left) and 1 min after probe infusion (right; neutrophils, red; thrombin activity, green). **(Aii)** Quantification of thrombin activity measured as a % of the FOV labelled by the fluorescent substrate in IAV-infected control and argatroban-pretreated animals. Quantification of neutrophils **(B)**, platelet aggregation **(C)**, extracellular histone **(D)**, BALF cellularity **(E)**, lung MPO activity **(F)**, lung wet-to-dry tissue weight ratios **(G)**, animal body mass **(H)** and viral loads **(I)** in IAV-infected control and argatroban-pretreated mice. Values represent the mean +/- SEM. n=4-8 per group. *p < 0.05, **p < 0.01, ***p < 0.001 compared to the control group. ^#^p < 0.05, ^##^p < 0.01 compared to the IAV-infected group.

It has been shown that NETs potentiate thrombin generation and drive intravascular coagulation in liver after S. aureus infection (31). To determine if a similar process occurs in the lung following IAV infection, we utilized a quenched-fluorescence peptide substrate to visualize thrombin activity within the living animal (31). Robust intravascular thrombin activity was observed within the lung microvasculature of IAV-infected mice (**Figure 4Ai**). To confirm thrombin specificity of the peptide probe, mice were given argatroban (a thrombin inhibitor), which demonstrated significant attenuation of fluorescence intensity (**Figure 4Aii**). Although inhibition of probe activation by argatroban does not completely rule out the possibility of another protease, this observation is strongly suggestive of a role for thrombin in the host-response to IAV infection. Interestingly, pretreatment of mice with argatroban significantly reduced neutrophil recruitment, platelet aggregation and NET formation 5 days after IAV infection (**Figures 4B**–**D**). Cell recruitment was confirmed by measuring the cellularity of the BALF and quantifying lung MPO activity (**Figures 4E, F**). Furthermore, pretreatment with argatroban also reduced wet-to-dry weight ratios and weight loss induced by IAV (**Figures 4G, H**). Some mice were treated with vehicle, DMSO, and no effect on weight loss, MPO activity or wet-to-dry weight ratios was observed (**Supplemental Figure 7**). Importantly, inhibition of thrombin did not exacerbate infection as the viral load recovered from the lungs 5 days post-infection was not changed (**Figure 4I**). These results demonstrate thrombin activation precedes NET generation in IAV-infected lungs and potentiates platelet aggregation, neutrophil recruitment, NET production, and tissue pathology in IAV-infected lungs but does not appear to play a role in viral control. Interestingly, argatroban had no effect if administered as a therapy at the time of neutrophil recruitment and NET generation (at day 3 and 4 of infection [**Supplemental Figure 8**]).

### PAR4 Contributes IAV-Induced Inflammatory Lung Tissue Damage

**Figure 5:**
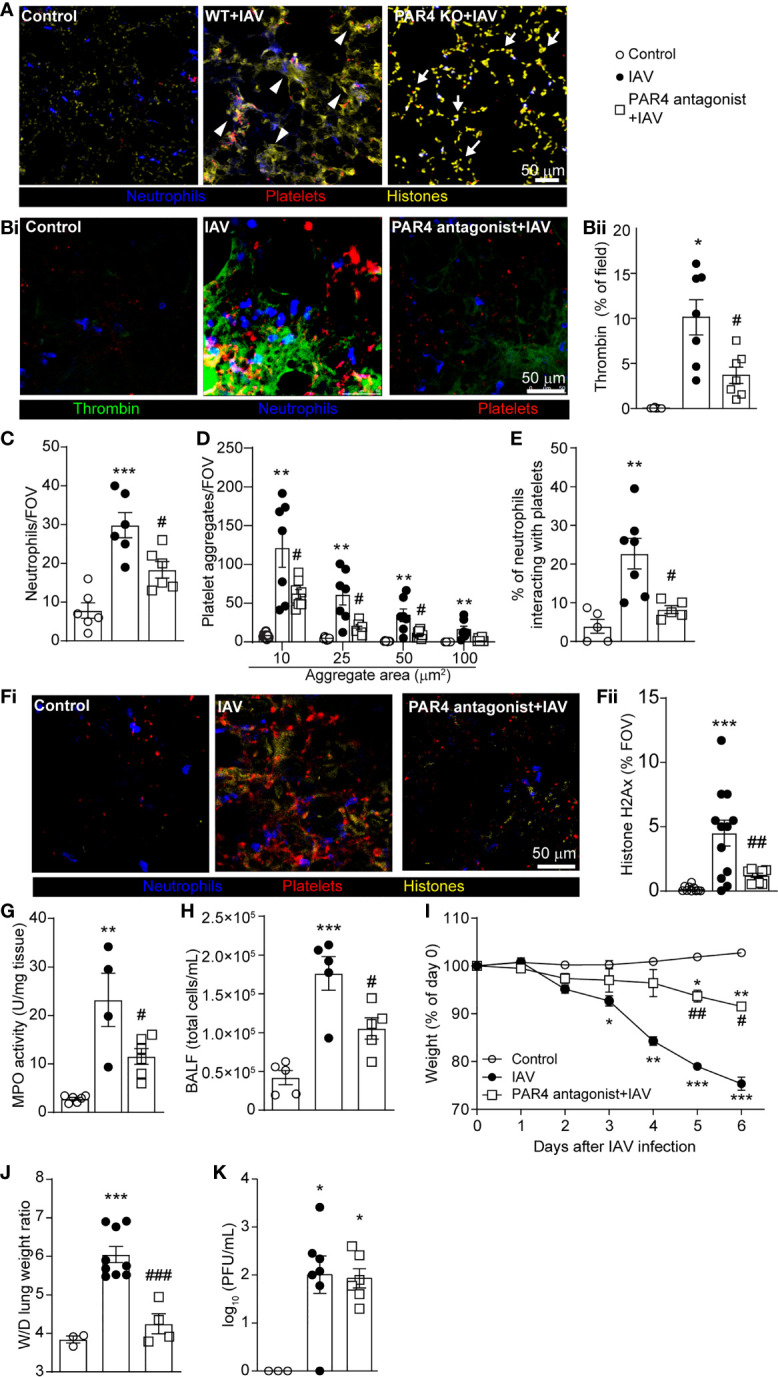
PAR4 Drives IAV-Induced Inflammation and Subsequent Tissue Damage. **(A)** Representative immunostaining of WT lung tissue uninfected control and WT and PAR4 KO (PAR4^-/-^) mice 5d after IAV infection (neutrophils, blue; histone, yellow; platelets, red). Arrowheads denote diffuse, intravascular histone staining (NETs), and full arrows indicate punctate, nuclear histone stain. **(Bi)** Representative IVM image of lungs of control (uninfected) and 5d post IAV infection of control and PAR4 antagonist (TcY-NH_2_)-pretreated mice demonstrating intravascular thrombin activity (neutrophils, blue; platelets, red; thrombin activity, green). **(Bii)** Quantification of thrombin activity measured as a % of the FOV labelled by the fluorescent substrate in uninfected control, IAV-infected control and PAR4 antagonist pretreated animals. Quantification of neutrophils **(C)**, platelet aggregation **(D)**, platelet-neutrophil interactions expressed as a % of neutrophils contacting platelets **(E)** in IAV-infected control and PAR4 inhibitor (TcY-NH_2_) pretreated mice. **(Fi)** Representative IVM image of lungs of control (uninfected) and 5d post IAV infection of control and PAR4 antagonist (TcY-NH_2_)-pretreated mice demonstrating histone staining (neutrophils, blue; histone, yellow; platelets, red). Quantification of extracellular histone **(Fii)**, lung MPO activity **(G)**, BALF cellularity **(H)**, animal body mass **(I)**, lung wet-to-dry tissue weight ratios **(J)**, and viral loads **(K)** in IAV-infected control and PAR4 inhibitor (TcY-NH_2_) pretreated mice. Values represent the mean +/- SEM. n = 4-12 per group. *p < 0.05, **p < 0.01, ***p < 0.001 compared to the control group. ^#^p < 0.05, ^##^p < 0.01, ^###^p < 0.001 compared to the IAV-infected group.

PAR4 is the primary thrombin receptor on mouse platelets and plays an essential role in platelet activation. Immuno-histochemistry on lung sections demonstrated an apparent increase in NET and/or extracellular DNA release (**Figure 5A**; diffuse, intravascular histone staining; arrowhead) and increased numbers of platelet aggregates in wild-type animals following IAV infection. Analysis of lungs from PAR4-deficient animals demonstrated increased staining for histone, likely reflecting the increased number of cells (and by extension nuclei) recruited to the IAV-infected lung, a reduction in diffuse NET-like staining was observed (histone remains as a punctate, nuclear stain; full arrow). As this staining was conducted on tissue sections obtained from frozen samples, we were unable to determine specifically the levels of extracellular versus intracellular (nuclear) histone but the difference in appearance (diffuse verses punctate) prompted us to investigate the possibility that PAR4 activation was needed for the production of NETs. To further explore the role of PAR4 in platelet aggregation and subsequent neutrophil activity, we administered a PAR4 peptide antagonist (trans-cinnamoyl-YPGKF-NH2 [TcY-NH2]) (47) prior to IAV infection. Pretreatment with PAR4 antagonist significantly attenuated intravascular thrombin formation following IAV infection (**Figure 5B**) indicating platelet activation, through PAR4, results in a positive feedback loop, augmenting thrombin production. In addition, pretreatment with PAR4 antagonist significantly reduced neutrophil recruitment, platelet aggregation and platelet-neutrophil interactions 5 days after IAV infection (**Figures 5C**–**E**, **Supplemental Video 4**). Inhibition of thrombin, or antagonism of PAR4 also reduced overall neutrophil activation and crawling (**Supplemental Figure 9**). In accordance with reduced neutrophil recruitment and platelet aggregation, NET release was also significantly diminished after PAR4 inhibitor treatment (**Figure 5F**) and PAR4 inhibition prior to IAV infection resulted in a significant reduction in MPO activity in the lung and BALF cellularity (**Figures 5G, H**).

PAR4 has been implicated in immune-mediated tissue damage, and it has been reported that PAR4 inhibition offers cytoprotection in sepsis and other inflammatory diseases (48, 49). Whereas IAV infection results in reproducible weight loss in mice, PAR4 antagonist-pretreatment significantly reduces weight loss after IAV infection (**Figure 5I**). Moreover, pretreatment with PAR4 inhibitor resulted in a reduced wet-to-dry weight ratio of lung tissue (**Figure 5J**) illustrating a role for PAR4 in lung pathology following severe IAV infection. Importantly, as observed with the inhibition of thrombin, antagonism of PAR4 had no impact on viral loads suggesting this pathway is not directly involved in pathogen clearance within the context of severe IAV infection (**Figure 5K**). These results suggest PAR4 is involved in IAV-induced inflammatory responses. Additionally, inhibition of PAR4 not only attenuates the amplification of this inflammatory response, but also limits the early steps of virus-induced pathology and prevents tissue damage in severe IAV infection.

## Discussion

Several studies have indicated an excessive inflammatory response to IAV infection is detrimental, worsening the severity of influenza pneumonia at multiple levels (14, 50). Uncontrolled inflammation is mediated by various cell lineages and the combination of multiple cytokines and other inflammatory mediators such as bioactive lipids. Platelets are important source of inflammatory mediators which can be rapidly released upon activation. Importantly, not only do platelets release cytokines and chemokines, but they also directly interact with inflammatory leukocytes and potentiate the effector responses of these cells (51). Furthermore, there is clear evidence of platelet activation in the blood of critically ill influenza patients (52), and platelets have been suggested to play an active role in development of pathogenesis during IAV infections (33). In animal models of IAV infection, H1N1 promotes platelet activation through the formation of immune complexes and thrombin generation (53). Considering the broad inflammatory role of activated platelets, including their direct release of microvesicles and indirect interaction with other inflammatory cells, platelet activation may represent a key contributor to the excessive inflammation frequently observed following viral infection. Using IVM, we directly visualized platelet recruitment, activation, and behavior within intact lung tissue during severe IAV infection.

Platelet-neutrophil interaction is a crucial process that drives immune responses during various models of inflammatory disease (54, 55). McDonald et al. (20) demonstrated that blocking platelet-neutrophil interactions improved sepsis-induced acute liver injury Moreover, it is known that platelets drive NET production (20, 56), facilitate neutrophil recruitment, and platelet-endothelial cell interactions drive lung injury during IAV infection (33, 34); however, the specific mechanisms involved in these interactions, and their role in IAV pathology have not yet been elucidated. In our study, we provide evidence that, within the context of severe IAV infection, platelet recruitment to, and aggregation within the lung vasculature precedes activation and recruitment of neutrophils. Large platelet aggregates form in lung vasculature within the first 24h following infection and subsequently neutrophils are recruited, co-localizing with aggregated platelets. These platelet-neutrophil interactions appear critical for the progression of lung pathology following IAV infection. Inhibition of platelet aggregation (CD41-deficient mice) or platelet-neutrophil interactions (CD18 blocking antibodies), prevents NET formation and subsequent lung tissue damage. Our findings demonstrate a requirement for platelet aggregation on adherent neutrophils for the generation of NETs, an effector mechanism that, when produced in excess, causes tissue injury (14). However, these findings must be balanced with the previous evidence that NETs released during infection can trap and inactivate viruses, potentially contributing to pathogen clearance *in vivo* (30, 57). Interestingly neither inhibition of NET formation (CD18 blockade/CD41 deficiency), nor removal of NETs from the vasculature (DNase), impacted viral clearance suggesting that within the context of severe IAV infection, NETs do more harm than good.

Following IAV infection, thrombin activates platelets, promoting both coagulation and the release of inflammatory microparticles from platelets (53). We hypothesized that an early thrombin signal generated shortly after severe IAV infection represents an inflammatory “spark” that bridges coagulation and inflammation, potentiating platelet-mediated inflammatory responses. Recent studies have pushed platelets into the forefront of IAV research, demonstrating that platelets amplify the host inflammatory response to IAV infection (33, 53, 58, 59). Importantly, using a mix of *in vitro* human and *in vivo* mouse studies, these previous studies confirmed that platelets can directly bind IAV virions and can initiate signaling through innate pattern recognition receptors (PRRs). These studies clearly illustrate the role platelets play in the host response to influenza and highlight innate immune receptors expressed by platelets.

It is important to note that PRRs are not the only means by which platelets can respond to an inflammatory signal. Through experiments that directly inhibit thrombin (argatroban), we identified a central role for thrombin in platelet activation and the initiation of inflammation in the lung following IAV infection. Not only was platelet aggregation and NET generation reduced in argatroban-pretreated animals, but a significant decrease in neutrophil recruitment and pathology was also observed following IAV infection in argatroban-treated animals demonstrating a requirement for thrombin-mediated signaling in the initiation of the host inflammatory response following severe viral infection.

NETs, and their components (DNA, histone proteins, neutrophil proteases) are one of the primary driving forces in the initiation of thrombosis and inhibition of fibrinolysis; hallmarks of disseminated intravascular coagulation (18, 60). These findings suggest a vicious cycle of NET release, microvascular thrombosis, thrombin-induced platelet activation which then precipitates further platelet aggregation, amplifying inflammation and tissue damage. Our data indicate that thrombin generation, within the context of IAV infection, precedes NET release and may represent a potential target in the mitigation of lung inflammation following viral infection.

PARs are receptors that facilitate thrombin-mediated signal transduction. Importantly, PAR1 and PAR4 are reported to be involved in development of IAV pathogenesis (33, 61–63). As mouse platelets do not express PAR1, thrombin-mediated platelet activation occurs through PAR4 (64, 65). Here, we demonstrate that inhibition of PAR4 drastically attenuates the formation of platelet aggregates, platelet-neutrophil aggregates, and reduces neutrophil recruitment following IAV infection. In addition, we show PAR4 inhibition attenuates subsequent thrombin generation and tissue pathology. In contrast, recent work by Tatsumi et al. (35) demonstrated that a global deficiency of PAR4 worsens disease in the context of mild to moderate IAV infection suggesting PAR4 may be protective during influenza infection. To reconcile our findings with these previous observations, we treated animals with a PAR4 antagonist followed by infection with a lower titer of IAV. Surprisingly, whereas PAR4 inhibition was protective when animals were challenged with a high viral load, inhibition of PAR4 in the context of a less severe infection worsened disease (**Supplemental Figure 10**), directly confirming the results of Tatsumi et al. It may be the case, that in the presence of low viral load, PAR4 signaling on non-platelets is required to increase the sensitivity of PRRs; however, in the case of higher viral loads, PRR sensitivity is no longer an issue and instead, PAR4 signaling, and activation of platelets becomes pathogenic. This creates an interesting dichotomy whereby the same receptor is both protective and pathogenic depending on the viral load, leading to potential problems with pursuing the PAR4-pathway as a therapeutic target for the treatment of IAV infection. Moreover, proteases released from neutrophils also have the ability to activate PARs, potentially bypassing the effect of anti-thrombin drugs and exacerbating the effect of the inflammatory milieu (66–68). These findings highlight the need for a better overall understanding of the overlapping inflammatory pathways at play in the lung before we can safely attempt to modulate thrombin generation or PAR activation in an effort to treat IAV infections.

Current approaches to treating IAV infection, focused on targeting specific viral proteins, have several disadvantages, including the rapid development of resistant/escape viral variants. Based on this very real limitation, drugs regulating the host inflammatory response to IAV infection might prove to be a more appealing target for future therapeutics. Treatment strategies that limit neutrophil recruitment, reduce NET production, or enhance NET clearance may effectively reduce lung pathology, although there is a risk that such treatments may also reduce the efficacy of the host immune response. Given the central role platelets play in the initiation and amplification of the host inflammatory response, thrombocytes represent an interesting potential target in the treatment of viral lung infections. Importantly, many clinically approved drugs that modulate platelet activation are already in use, making the development of potential therapeutic strategies a realistic possibility. Our results highlight dynamic and coordinated innate immune responses within lung vasculature during IAV infection. We demonstrate that modulating platelet activation, and their interactions with other immune cells, may protect lungs from IAV-induced pathogenesis in an experimental model of viral infection. Furthermore, we also provided insight on infection-induced thrombin, with evidence suggesting thrombin is a key molecule linking coagulation and inflammation, both of which are crucial axes in the immunopathology, and resolution, of IAV infection.

## Data Availability Statement

The raw data supporting the conclusions of this article will be made available by the authors, without undue reservation.

## Ethics Statement

The animal study was reviewed and approved by University of Calgary Animal Care Committee.

## Author Contributions

S-JK and AC designed and performed experiments, analyzed data, and prepared figures. HG, AZZ, RD, MT, and VN performed experiments and analyzed data. SA performed experiments and contributed reagents/materials/analysis tools. NM and MFA-C contributed reagents/materials/analysis tools. MSA-C performed experiments, analyzed data and contributed reagents/materials/analysis tools. MDH designed experiments and contributed reagents/materials/analysis tools. CNJ designed experiments and together with S-JK and BM wrote the paper. All authors contributed to the article and approved the submitted version.

## Funding

This research was supported by Basic Science Research Program through the National Research Foundation of Korea (NRF) funded by the Ministry of Education (2014R1A6A3A03058770) and through grants from the Heart and Stroke Foundation of Canada (HSFC), the Natural Sciences and Engineering Research Council of Canada (NSERC), the University of Calgary URGC, the Lung Association of Alberta & NWT, the Canadian Foundation for Innovation, Alberta Innovates and Advanced Education. CJ is supported by the Canada Research Chairs program.

## Conflict of Interest

The authors declare that the research was conducted in the absence of any commercial or financial relationships that could be construed as a potential conflict of interest.

## Publisher’s Note

All claims expressed in this article are solely those of the authors and do not necessarily represent those of their affiliated organizations, or those of the publisher, the editors and the reviewers. Any product that may be evaluated in this article, or claim that may be made by its manufacturer, is not guaranteed or endorsed by the publisher.
