# Supplementary material for: Platelet-Mediated NET Release Amplifies Coagulopathy and Drives Lung Pathology During Severe Influenza Infection
Source: Front Immunol. 2021 Nov 11;12:772859. doi: 10.3389/fimmu.2021.772859 (PMC8632260; doi:10.3389/fimmu.2021.772859)
Supplement: Supplementary file 1 [file DataSheet_1.pdf]

Supplementary Material

1 Supplementary Figures

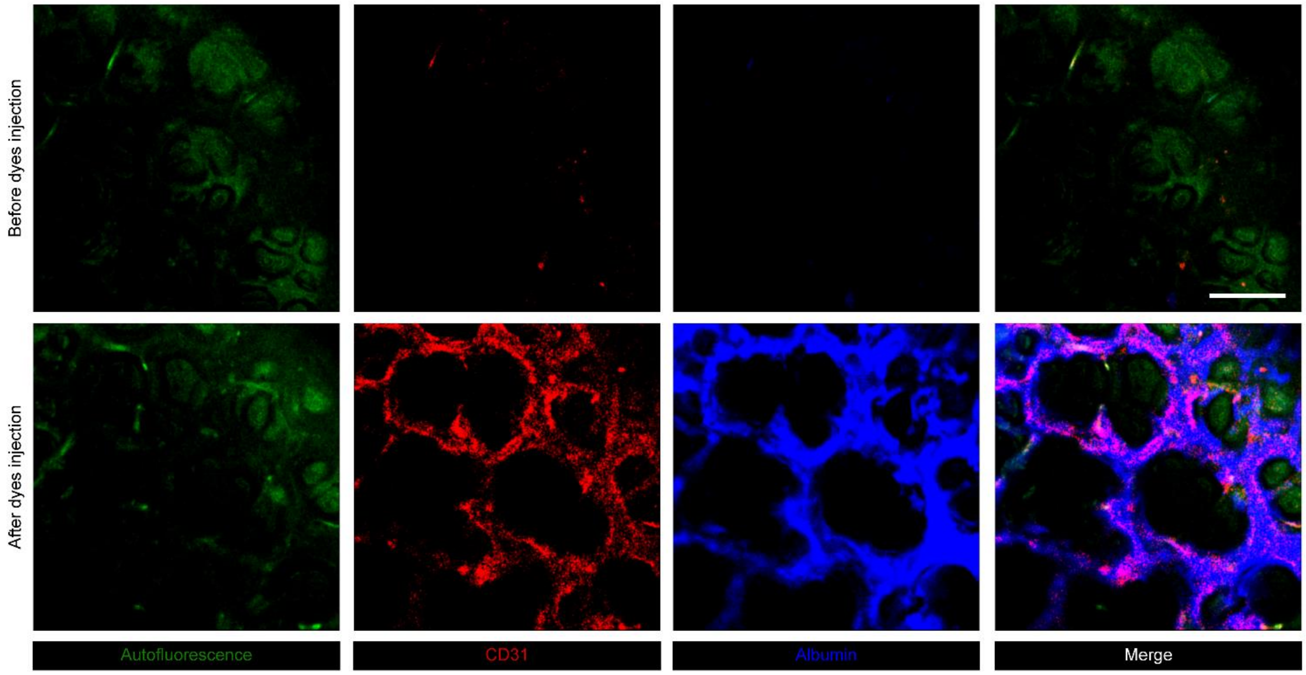

Supplemental Figure 1. IVM image of a control lung. Intravital visualization the lung of a control mouse. Top row of images obtained from the animal prior to the introduction of any fluorescent labels demonstrating the natural autofluorescence of the tissue. Bottom row of images illustrates the labeling of the vasculature surrounding the alveolar spaces (CD31; red). Introduction of fluorophore-conjugated albumin (blue) highlighting blood flow and confirming the absence of fluorophore leakage from the vasculature into the airspaces during the imaging process.

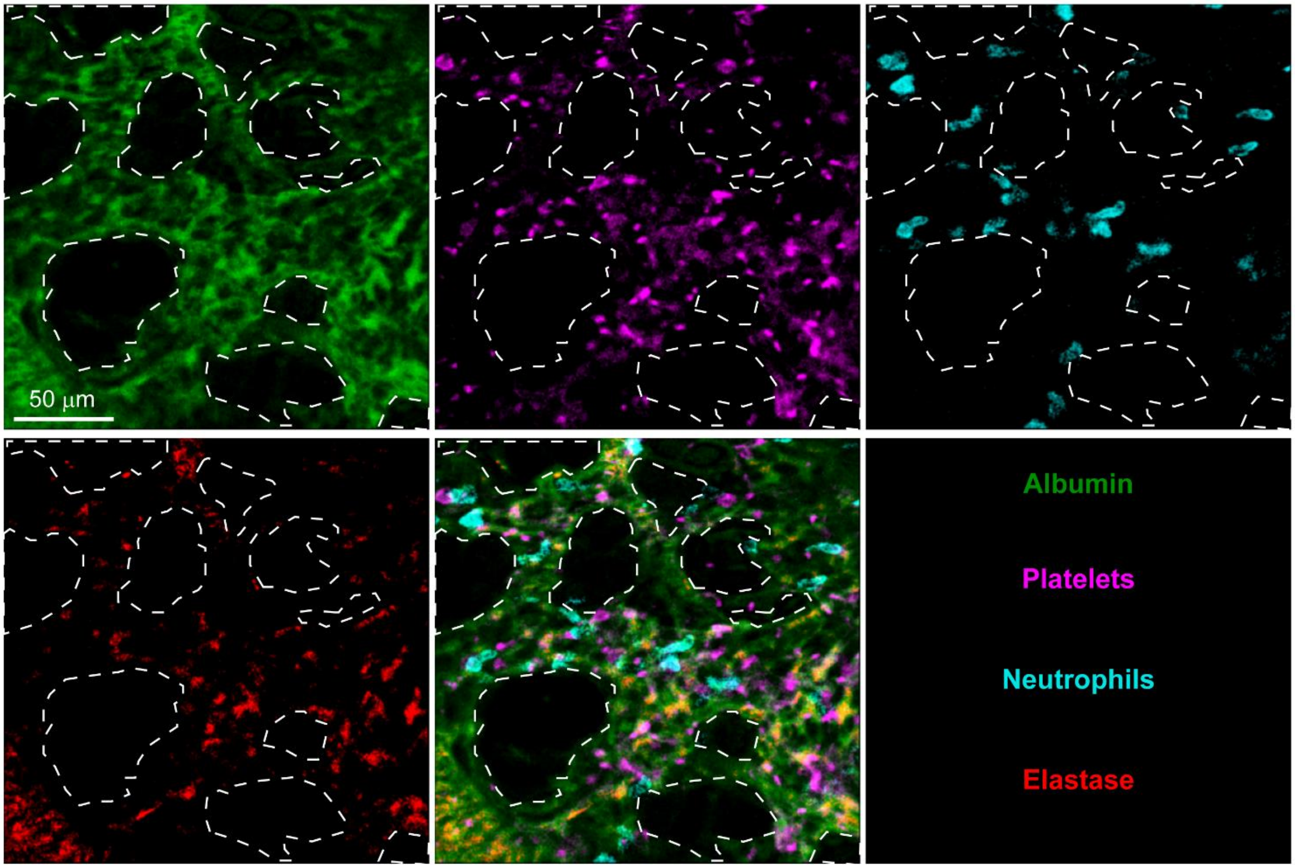

Supplemental Figure 2. IVM image of the lung after 5 days of IAV infection. Intravital visualization of albumin, platelets, neutrophils, and neutrophil elastase following infection with IAV in the lung vasculature. Dotted lines delimit the alveolar spaces.

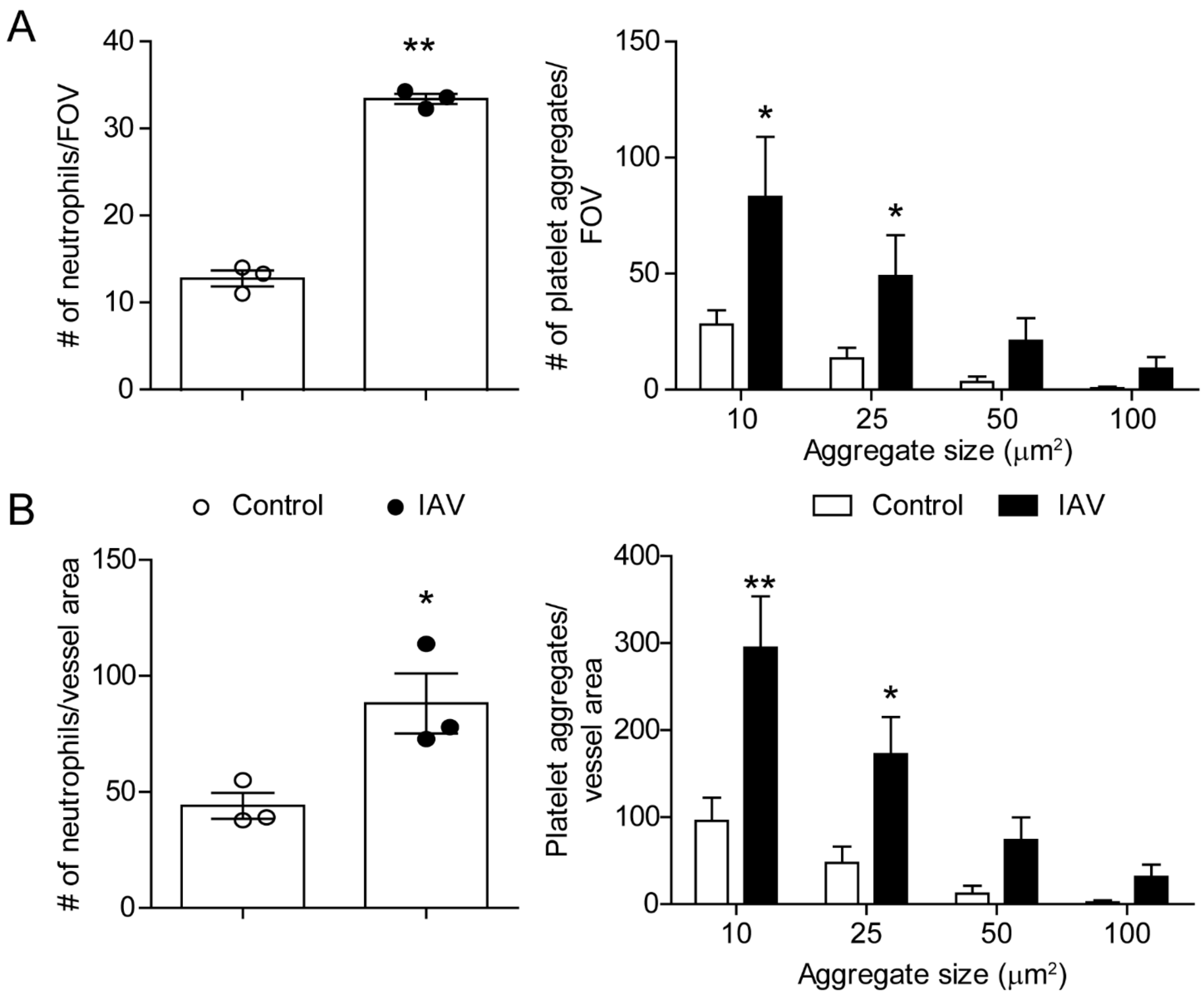

Supplemental Figure 3. Comparison of the analysis of cells by field of view (FOV) and vessel area. Quantification of neutrophils and platelet aggregates within the lung measured by IVM per FOV **(A)** or vessel area **(B)**. Values are the mean number of neutrophils or aggregates per field of view (FOV) or vessel area ± SEM, n=3 per group. * $p < 0.05$, ** $p < 0.01$ compared to the control group.

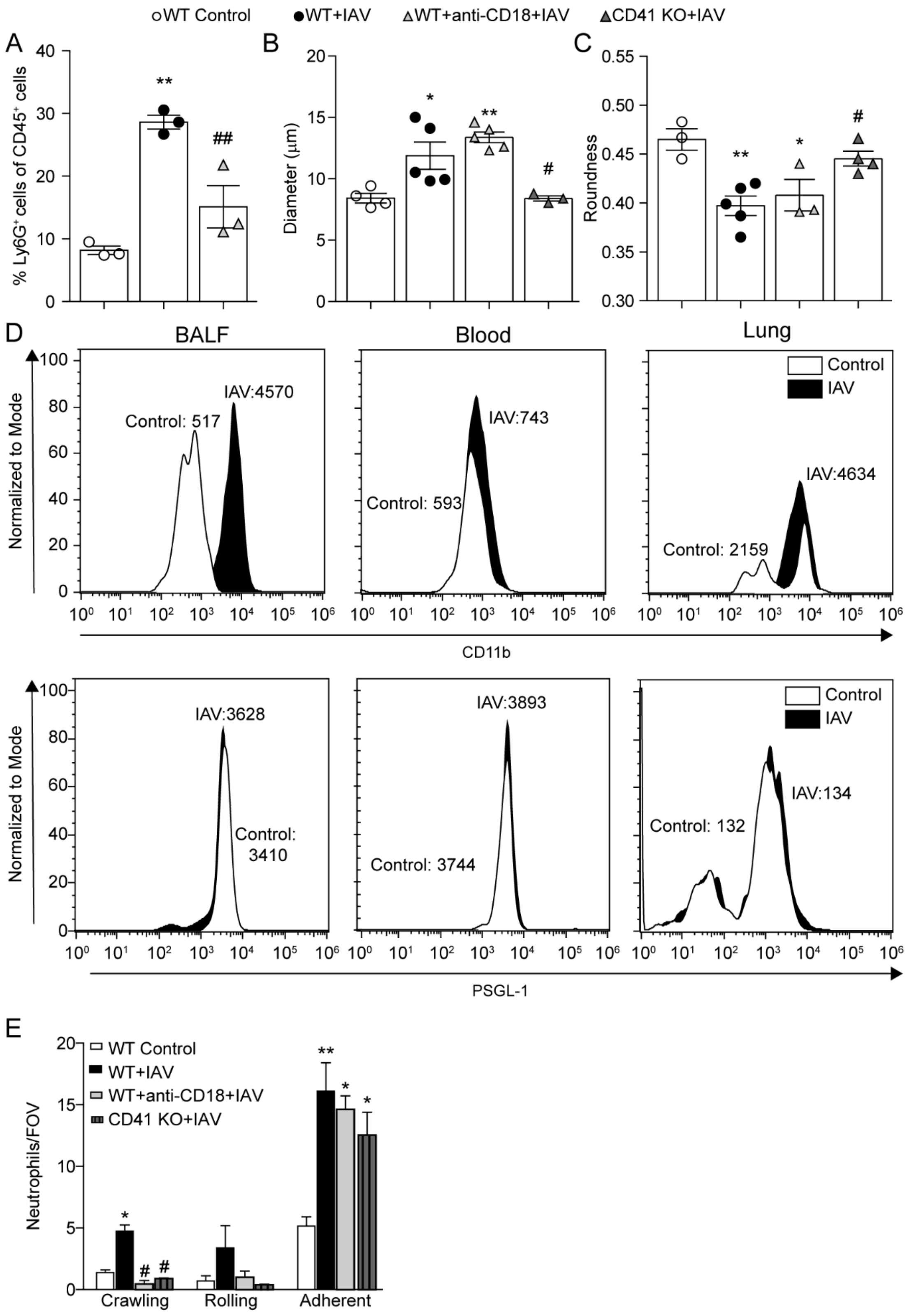

Supplemental Figure 4. Assessment of Neutrophil Activation by IVM in IAV-infected Mice Treated with Anti-CD18 Blocking Antibodies or Deficient for CD41. (A) Flow cytometric

quantification of the percentage of neutrophils (Ly6G$^+$ cells) in the lung. Measurement of neutrophil diameter **(B)** and cellular roundness **(C**; 1 = perfect circle) in control, IAV-infected, anti-CD18 treated IAV-infected and IAV-infected CD41-deficient animals. **(D)** Representative histograms of CD11b and PSGL-1 expression in neutrophils measured by flow cytometry in BALF, blood and lung in control and IAV-infected mice **(E)** Neutrophil behaviour (crawling, rolling, and adherent) in control, IAV-infected, anti-CD18 treated IAV-infected and IAV-infected CD41-deficient animals. Rolling was defined as a discrete neutrophil interaction with the vascular wall, which arrests its circulatory movement for less than 30 s. Crawling was defined as continuous interaction of a neutrophil with the vascular wall for more than 30 s, which involves a polarized cell that does not remain stationary. Adherent was defined as a stationary neutrophil that was not mobile and remained static for at least 30 s. Values represent the mean ±SEM. n=3-5 per group. *p < 0.05, **p < 0.01 compared to control; #p < 0.05 compared to IAV-infected.

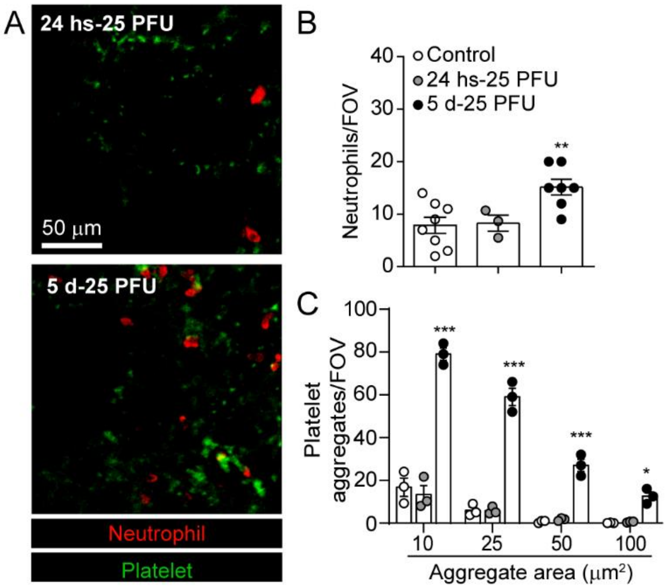

Supplemental Figure 5. Neutrophil and Platelet Recruitment to the Lung 1 and 5-days Following IAV Infection with a Low Inoculum. (A) IVM of neutrophil recruitment and platelet aggregation 24h-post low dose (25 PFU) IAV infection and 5d-post low dose (25 PFU) IAV infection (neutrophils, red; platelets, green). **(B)** Quantification of neutrophils within the lung as measured by IVM at different times post low dose (25 PFU) IAV infection. Values are the mean number of neutrophils per field of view (FOV) ± SEM, n=3-8 per group. **(C)** Quantification of platelet aggregates of indicated sizes at indicated times post low dose (25 PFU) IAV infection. Values are the mean number of aggregates per field of view (FOV) ± SEM, n=3-5 per group. * $p < 0.05$, ** $p < 0.01$, *** $p < 0.001$ compared to the control group.

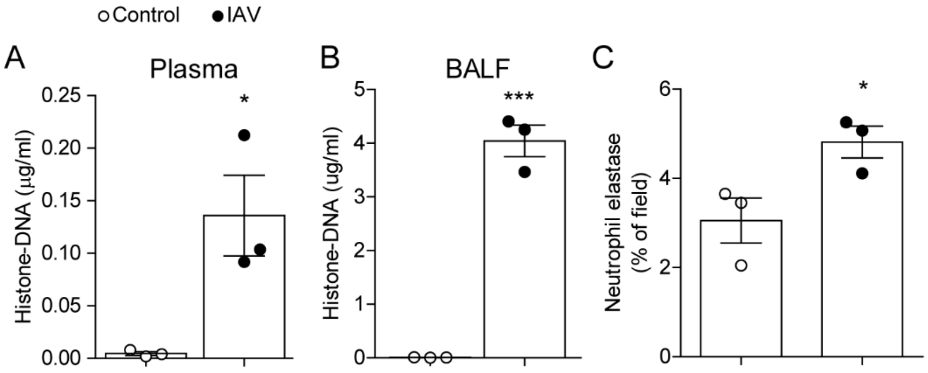

Supplemental Figure 6. Quantification of Histone-DNA and neutrophil elastase in control and IAV infected mice. Quantification of Histone-DNA complexes in plasma **(A)** and BALF **(B)** in control and IAV-infected mice measured by ELISA. **(C)** Quantification of the area of staining of neutrophil elastase measured by IVM. Values represent staining area as a % of the FOV and are presented as the mean+/- SEM. n=3 per group. * $p < 0.05$, ***$p<0.001$ compared to the control group.

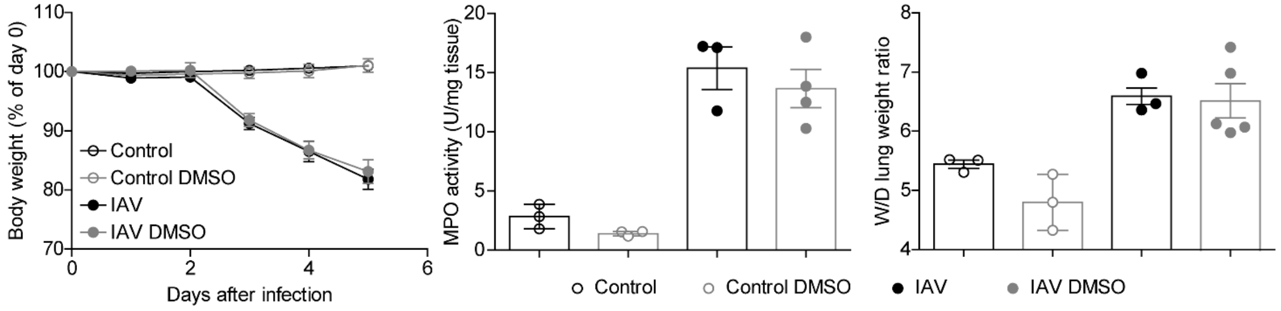

Supplemental Figure 7. Effect of DMSO (vehicle for argatroban) in control and IAV-infected mice. Mice were pre-treated with DMSO and infected with IAV. Weight loss **(A)**, MPO activity **(B)** and wet-to-dry weight ratio **(C)** were measured. Values represent the mean ±SEM. n=3-4.

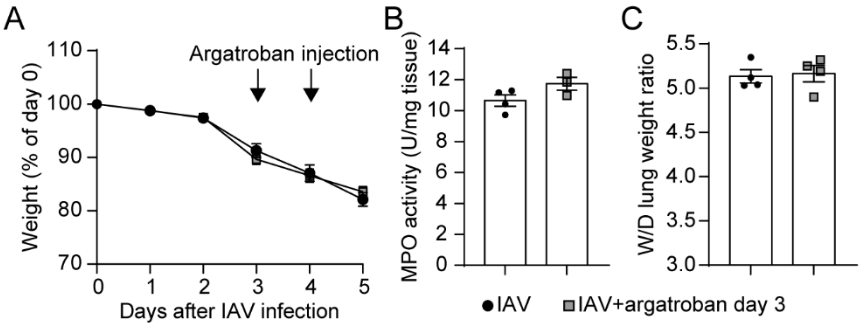

Supplemental Figure 8. Administration of argatroban after IAV infection does not protect mice. Animal body mass **(A)**, lung MPO activity **(B)**, and lung wet-to-dry tissue weight ratios **(C)**, in IAV-infected mice and mice that were administrated argatroban on day 3 and day 4 post infection. Values represent the mean ±SEM. n=3-4 per group.

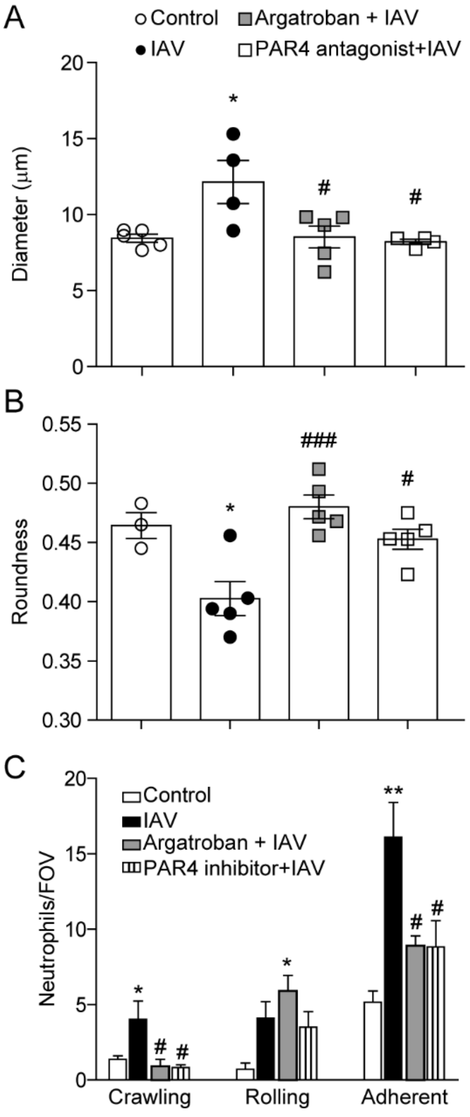

Supplemental Figure 9. Assessment of Neutrophil Activation by IVM in IAV-infected Mice Treated with DNase, Argatroban or PAR4 Antagonist. Measurement of neutrophil diameter **(A)**, cellular roundness **(B)** and neutrophil behaviour (crawling, rolling, and adherent) **(C)** in control, IAV-infected, argatroban-treated IAV-infected, or PAR4 antagonist-treated IAV-infected animals. Values represent the mean ±SEM. n=3-5 per group. *p < 0.05, **p < 0.01 compared to control; #p < 0.05, ###p <0.001 compared to IAV-infected.

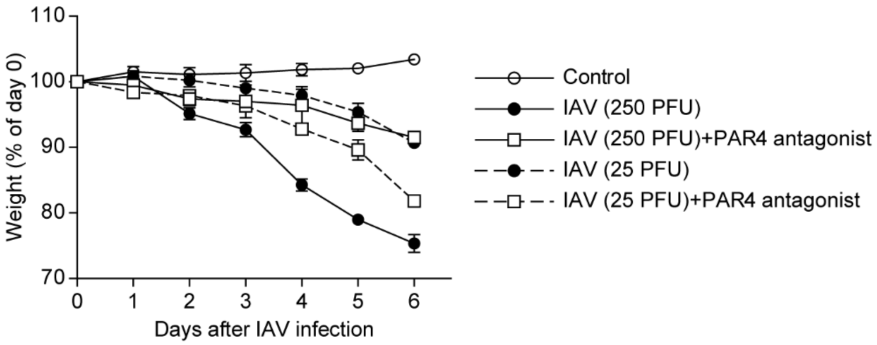

Supplemental Figure 10. Impact of PAR4 Antagonism in Response to Different Levels of Viral Challenge. Animal body mass assessments following infection with two different viral inoculums of PR8 IAV. In some cases, animals were pretreated with a PAR4 antagonist (TcY-NH2). Values represent the mean +/- SEM. n = 4-6 animals per group.

2 Supplementary videos

Supplemental Video 1. Increased Neutrophil Recruitment after IAV Infection in vivo. Intravital visualization of a lungs from a C57Bl/6 mouse 5 days after IAV infection (250 PFU i.n.). Neutrophil labelling of lung was achieved by intravenous injection of BV421-conjugated anti-Ly6G (red).

Supplemental Video 2. Platelet Aggregate Formation and Neutrophil Recruitment within the Lungs of an IAV-infected Mice. Intravital visualization of a lungs from a C57Bl/6 mouse 5 days after IAV infection (250 PFU i.n.). Platelets are labelled with AF647-conjugated anti-CD49b (green), and neutrophils are labelled with BV421-conjugated anti-Ly6G (red). Vascular labeling is achieved by injecting PE-conjugated anti-CD31 (white) in control mouse or FITC-conjugated albumin (white) in IAV challenged mouse.

Supplemental Video 3. Platelet Aggregation, Neutrophil Recruitment and Platelet-Neutrophil Interaction within the Lungs of Integrin Blocked/Deficient Mice after IAV Infection. Intravital visualization of a lungs from in WT (control), anti-CD18 antibody [i.v. 1 h prior to poly(I:C) or IAV challenge]-treated WT, and CD41 KO mice followed by i.t. poly(I:C) instillation (24 h) or i.n. IAV infection (5 days post infection). Platelets are labelled with AF647-conjugated anti-CD49b (green), and neutrophils are labelled with BV421-conjugated anti-Ly6G (red).

Supplemental Video 4. Visualization of Lung Neutrophils and Platelets on PAR4-Pretreated Mice. Intravital visualization of the lungs of PAR4 antagonist (TcY-NH2) or PBS (control)-pretreated and mice 5 days after IAV infection (250 PFU i.n.). Platelets are labelled with AF647-conjugated anti-CD49b (green); neutrophils labelled with BV421-conjugated anti-Ly6G (red).
